# Antioxidant, aroma, and sensory characteristics of Maillard reaction products from *Urechis unicinctus* hydrolysates: development of food flavorings

**DOI:** 10.3389/fnut.2024.1325886

**Published:** 2024-02-06

**Authors:** Mengdi Du, Wei Yu, Ning Ding, Mengqi Jian, Yongqiang Cheng, Jing Gan

**Affiliations:** ^1^College of Life Science, Yantai University, Yantai, Shandong, China; ^2^Beijing Key Laboratory of Functional Food from Plant Resources, College of Food Science and Nutritional Engineering, China Agricultural University, Beijing, China

**Keywords:** *Urechis unicinctus*, the Maillard reaction, ROS, antioxidation, functional condiment

## Abstract

To develop food flavorings with a delicious taste and an anti-oxidation effect, in this study, the glucose Maillard reaction was used for hydrolysates of *Urechis unicinctus*. The various biological activities of Maillard reaction products (MRPs) and their antioxidant capacity were evaluated. The results showed that the unique fishy odor substances of seafood in MRPs were reduced, indicating that the Maillard reaction improved the flavor of the hydrolysate of *Urechis unicinctus*. Meanwhile, MRPs exhibited more competitive radical scavenging activities compared to the hydrolysate. Moreover, MRPs demonstrated a considerable potential to protect against 2,2′-Azobis (2-methylpropionamidine) dihydrochloride (AAPH)-induced oxidative stress in a cell model *in vitro* and in a zebrafish model *in vivo*. Finally, a novel food flavoring was produced with MRPs as raw material, while the sensory qualities were deemed acceptable. In consequence, during industrial production, MRPs of *Urechis unicinctus* hydrolysate act as a high-quality raw material for functional flavorings and provide an effective way for the utilization of marine resources.

## 1 Introduction

Flavorings are usually food ingredients that are used to be added in small quantities to other foods to improve their flavor (deodorize, enhance freshness, relieve grease, etc.) (1). The flavoring industry has a wide range of products and a large sales volume, especially in China and other south-central Asian countries. The flavoring industry is developing rapidly, and the annual production of flavoring can be more than ten million tons. Especially in recent years, with the rapid development of society, people’s living standards are improving at lightning speed, and the majority of consumers on the requirements for seasonings are also on the rise. Compared with the popular flavorings sold in the market now, people’s requirements for flavorings are not only reflected in the color and flavor but also in being natural and environmentally friendly, green and safe, with specific nutritional properties. Therefore, seasonings with certain functions have become a hot spot for research in the food industry and the mainstream development direction of China’s seasoning industry. Aquatic seasoning is becoming more and more popular because it retains the original unique flavor of aquatic products (2), and contains rich amino acids, nucleotides, small peptides, and other active ingredients, and it has shown a rapid growth trend in international market demand. The production and consumption of aquatic seasonings in foreign countries are mainly distributed in Southeast Asia, Japan, and the northern Philippines. At present, seafood seasonings account for about 60% of the Japanese seasoning market, and gradually replace traditional seasonings such as monosodium glutamate in Southeast Asia. Aquatic seasoning has gradually become the mainstream development direction of international condiments. China is rich in marine resources, and the development of functional marine food is the strategic goal of our country’s future development. Making full use of marine animal protein resources is an urgent problem for the aquatic product processing industry.

*Urechis unicinctus*, also known as the sea intestine, is a long cylindrical annelid. Its protein content is rich, accounting for about 71.01% of its dry weight, which is much higher than that of other aquatic products (mussel protein accounted for 58.5% and oyster protein accounted for 47.21%), and it also contains peptides, polysaccharides, and other physiologically active substances (3), which have the hypoglycemic effect, antihypertensive and antioxidant activity (4, 5). The muscle hydrolysate of *Urechis unicinctus* contained 18 amino acids, comprising five types of umami amino acids and eight types of necessary amino acids. The composition of amino acids was reasonable, and the umami amino acids representing 53.63% of the total amino acids. Therefore, *Urechis unicinctus* is an excellent source for the development of functional condiments. What’s more, the processing and utilization of *Urechis unicinctus* in China and other Southeast Asian countries is still in its infancy, mainly in the form of cooking. There are relatively few studies on flavor, especially in the development of seasonings. The development of seafood seasoning is one of the effective way to realize the high value utilization of marine resources, so the preparation of *Urechis unicinctus* seafood seasoning products has become the trend of the *Urechis unicinctus* industry. However, in general, as the freshness of seafood decreases, it will produce a unique smell, which may have a negative impact on the flavor of *Urechis unicinctus*. Therefore, improving this bad flavor is a prerequisite for the development of *Urechis unicinctus* seasoning. There are many methods to improve this unpleasant odor, such as adsorption, extraction, biological deodorization, and the Maillard reaction (MR), among which MR method is deeply favored by the food industry. The use of MR technology to treat the protein hydrolysate of aquatic products is a simple and low-cost process, and the purpose of deodorization and flavor enhancement can be achieved at the same time.

The Maillard reaction (MR), additionally referred as non-enzymatic browning, is a non-enzymatic reaction that takes place between the carbonyl group of reducing sugar and the amino group of amino acids, peptides, or proteins (6, 7), which can improve the disagreeable taste of food (8–12). The Maillard reaction has been extensively used in food processing, flavor chemistry, and other realms (13–15) and is essential for increasing the flavor and color of food products, resulting in distinctive color and flavor in pastry, cocoa bakery, and meat processing products (16). The Maillard reaction can not only improve flavor but also effectively improve antioxidant activity (17). For instance, Guérard et al. (18) reported that when casein peptone and cod offal hydrolysates were heated in the presence of glucose, the free radical scavenging effect increased by 75%. Some researchers have broadly divided the factors influencing the change in antioxidant activity into two categories: the type of reactants and the generation of some reaction products. Firstly, Hwang et al. (19) studied MRPs of more than 20 amino acids under the same conditions and found that MRPs produced by alanine, tyrosine, tryptophan, and asparagine had stronger free radical scavenging ability. Second, some antioxidant compounds are formed after the Maillard reaction, such as phenolics and acids (20–22). Consequently, we attempted to enhance the flavor and antioxidant capacity of the hydrolysate of *Urechis unicinctus* by means of the Maillard reaction, which supplied a foundation for the development of functional seasonings.

In this research, the enzymatic solution of *Urechis unicinctus* was reacted with glucose at 120°C for 2 h to prepare Maillard reaction products (MRPs). Compared to the hydrolysates, the components of MRPs may change. To evaluate these changes, high-performance liquid chromatography (HPLC) and gas chromatography–mass spectrometry (GC–MS) were utilized to detect the amino acid composition and flavor substances in the hydrolysates and MRPs (23). The antioxidant property of MRPs was evaluated by chemical approaches as well as the construction of L_02_ cells and zebrafish oxidative stress models. In addition, functional seasonings were prepared based on the Maillard reaction products of *Urechis unicinctus* hydrolysates.

## 2 Materials and methods

### 2.1 Materials

*Urechis unicinctus* were obtained from Yantai Laishan District Aquatic Products Market (Shandong, China). Fetal bovine serum (FBS) was acquired from Zhejiang Tianhang Biotechnology Co., Ltd. (Zhejiang, China). 3-(4,5-dimethyl-2-thiazolyl) -2,5-diphenyl-2H-tetra-zolium bromide (MTT), dimethyl sulfoxide (DMSO), Acridine Orange, 1,3-bis (diphenylphosphine) propane (DPPP), and 2,2′-Azobis (2-methylpropionamidine) dihydrochloride (AAPH) were procured from Sigma-Aldrich (St Louis, MO, USA). Amino Acids Mixture Standard Solution was obtained from KGaA (Darmstadt, Germany). GSH assay kits and MDA assay kits were bought from Nanjing Jiancheng Bioengineering Institute (Nanjing, China). BCA Protein Assay Kit was supplied by the Beyotime Institute of Biotechnology (Beijing, China). Dulbecco’s Eagle’s medium (DMEM), phosphate-buffered solution (PBS, pH 7.2–7.4, 0.01 M), L-ascorbic acid, 2′,7′-dichlorodihydrofluorescein diacetate (DCFH-DA), HO• scavenging activity assay kit, DPPH• scavenging activity assay kit, ABTS• scavenging activity assay, and alkaline protease were procured from Solarbio Science & Technology Co., Ltd. (Beijing, China). Pepsin was obtained from Macklin Biochemical Co., Ltd. (Shanghai, China). Trypsin, papain, and flavourzyme were acquired from Aladdin Reagent Co., Ltd. (Shanghai, China). Salt, sodium glutamate, disodium nucleotide, yeast extract, and white granulated sugar were obtained from Yantai New world Department Store (Shandong, China).

### 2.2 Preparation of enzymatic hydrolysate of *Urechis unicinctus*

After the viscera were removed, the fresh *Urechis unicinctus* was washed and stored in a refrigerator at −80°C. The cryopreserved *Urechis unicinctus* was placed in a freezing dryer. After 24 h, it was ground and crushed (24). A measured amount of distilled water was added and the pH was adjusted. After the hydrolysis was completed, the enzyme was killed at 95°C, cooled, centrifuged, and the supernatant was taken. The free amino acid was measured by the neutral formaldehyde method, and the total nitrogen content was detected by the Kjeldahl method (25). Based on the degree of hydrolysis, three proteases with the highest degree of hydrolysis were selected. Using the software Design-Expert1, the Simplex lattice experimental design method was utilized to identify the optimal ratio of compound protease. Then the optimal ultrasonic conditions and enzymatic hydrolysis conditions (4) were optimized by single factor test and response surface experiment. The enzymatic hydrolysate prepared under the optimal conditions was freeze-dried and placed in a refrigerator at −20°C for subsequent use.

### 2.3 Preparation of Maillard reaction products (MRPs)

Under the optimal conditions MRPs of the hydrolysate of *Urechis unicinctus* were prepared from the lyophilized powder of the enzymatic hydrolysate. The powder (10 g) was solved in 100 mL of water at a proportion of 1:10 (w/v), and then the glucose (4 g) was incorporated into the Maillard reaction mixture. Followed by heating at 120°C for 2 h (26), the samples were promptly freeze-dried and kept at −20°C for subsequent use.

### 2.4 Bioactive compound analysis of Maillard reaction products

#### 2.4.1 Amino acid composition analysis of MRPs

The composition of amino acids was tested by HPLC utilizing a C18 column (5 μm, 4.6 × 250 mm). With this method, the composition of amino acids was analyzed separately for the Maillard reaction solution and the enzymatic digestion solution. The chromatographic conditions were as follows: Test wavelength was 254 nm, determination of column temperature was 40°C, injection volume was 10 μL, mobile phase: A phase was 0.1 mol/L sodium acetate solution (pH 6.50): acetonitrile = 93: 7, B phase was water: acetonitrile = 20: 80, gradient elution procedure: 0–0.01 min, 100% A; 0.01–35 min, 100 ~ 0% A; 35–42 min, 100% B; 42–45 min, 0–100% A, flow rate was 1.0 mL/min, and the total elution time was 60 min.

#### 2.4.2 Analysis of flavor substances of MRPs By GC–MS

The changes in flavor substances of the hydrolysate and MRPs were determined separately by GC–MS. The samples were subjected to solid-phase microextraction at 80°C for 40 min and sorption at 250°C for 3 min, which were tested afterwards. The GC–MS analysis was carried out on a DB-5MS column under the following conditions: The temperature of the injection port was 250°C, the split ratio was 10:1, and the carrier gas was high-purity helium 6.0 flowing at a rate of 1 mL/min. The temperature program was adapted as follows: maintain 40°C for 2 min, rise to 200°C at 6°C/min, then rise to 300°C at 15°C/min for 2 min. The temperature of the ion source was 220°C, the surface temperature was 280°C, and the m/z scan range was 33 to 500 for the full scan.

### 2.5 Determination of antioxidant activities with chemical methods

#### 2.5.1 DPPH radical scavenging activity

The method of Li et al. (27) was utilized to evaluate the DPPH radical scavenging capacity of hydrolysates and MRPs with minor adjustment. DPPH working solution is obtained by dissolving DPPH powder in ethanol. The 20 μL sample was combined with 180 μL DPPH working solution (0.2 mM) and incubated in darkness at room temperature for 30 min. Subsequently, the absorbance of the mixed solution was detected at 517 nm utilizing a microplate with L-ascorbic acid as a positive control. The DPPH free radical scavenging rate is calculated as follows:

DPPH radical scavenging activity (%) = {[A_blank_ − (A_sample_ − A_control_)]/ A_blank_} × 100%.

#### 2.5.2 ABTS radical scavenging activity

A minor modification was adapted to the procedure reported by Tan et al. (28) for sake of assessing ABTS radical scavenging capacity. The ABTS*^+^* solution was formulated by combining 2.45 mM potassium persulfate solution and 7 mM ABTS mother liquor in an equal volume, which was then incubated for 12–16 h at room temperature away from light. PBS was applied to dilute the solution to an absorbance of 0.70 ± 0.02 at 734 nm, and the ABTS working solution was obtained. Subsequently, 200 μL of ABTS working solution was combined with 8 μL of the sample solution. The mixture was incubated under darkness at room temperature for 10 min before the absorbance was tested at 734 nm with L-ascorbic acid as the positive reference. The results of the calculations are expressed as the percentage of the scavenging rate using the following formula:


$$\begin{array}{l} \text{ABTS}\;\text{radical}\;\text{scavenging}\;\text{activity}\;\left(\%\right)=\\ \left\{\left[A_{\text{blank}}-\left(A_{\text{sample}}-A_{\text{control}}\right)\right]/A_{\text{blank}}\right\}\times 100\% \end{array}$$

#### 2.5.3 HO radical scavenging activity

HO• scavenging activity was determined with reference to the testing means used by Liu et al. (29) with light revision. The HO• radical was produced by FeSO*_4_* and H*_2_*O*_2_*, which was detected by its ability to hydroxylate salicylate. The reaction system (0.2 mL) included 50 μL FeSO_4_ (9 mM), 50 μL H_2_O_2_ (9 mM), 50 μL sodium salicylate (9 mM), and 50 μL different concentrations of hydrolysate or MRPs. After 1 h of incubation at 37°C, the absorbance at 520 nm was tested, and ascorbic acid was utilized as a positive control. The following is how the percentage elimination effect was computed:


$$\begin{array}{l} HO\;\text{radical}\;\text{scavenging}\;\text{activity}\;\left(\%\right)=\\ \left\{\left[A_{\text{blank}}-\left(A_{\text{sample}}-A_{\text{control}}\right)\right]/A_{\text{blank}}\right\}\times 100\% \end{array}$$

### 2.6 To evaluate the antioxidant effect of MRPs in the L_02_ cell model

#### 2.6.1 Cell culture

L_02_ cells were cultivated in DMEM with 1% antibiotics (100 U/mL penicillin and 100 g/mL streptomycin) and 10% fetal bovine serum (FBS) at 37°C in a moist environment with 5% CO_2_. 0.25% trypsin was applied to subculture the cells when their confluence reached 80–90%.

#### 2.6.2 Cell viability assay

The viability of L_02_ cells induced by AAPH was assessed by the MTT method. The cells (1 × 10^5^/mL) were inoculated into 96-well plates and cultivated for 24 h. After that, the medium was renewed, and the cells were cultured in various doses of MRPs for 1 h. Afterwards, the cells were treated for 16 h in the absence or presence of AAPH (15 mM) (30). The final step was to incorporate 10 μL MTT solution (5 mg/mL). After 3 h of incubation, the purple formazan crystals were resolved in dimethyl sulfoxide (150 μL). With the use of a microplate reader (Molecular Devices, San Jose, CA, USA), the absorbance was measured at 570 nm.

#### 2.6.3 Determination of ROS levels

Cells were inoculated in 96-well plates at a density of 1 × 10*^4^* per well and grown for 24 h. Cells were subsequently recieved treatment with AAPH (15 mM, 16 h) and pre-incubated with MRPs for 1 h. After that, according to the instructions for the ROS detection kit, the cells were covered with DCFH-DA (10 mM). After 30 min, the intracellular ROS content was assessed by quantifying the fluorescence emitted by DCFH-DA oxidation to 2′, 7′-dichlorofluorescein. A microplate reader was applied to examine the fluorescence intensity at wavelengths of 485 nm for the excitation and 525 nm for the emission.

### 2.7 Antioxidant evaluation of MRPs in oxidative stress model *in vivo*

To estimate the antioxidative effect of the MRPs, we constructed a zebrafish model of oxidative stress. Zebrafish embryos were cultivated in 6-well plates. The 24 hpf zebrafish embryos were firstly treated with various doses of MRPs (0, 25, 50, 100, 200, 400 μg/mL) with the aim of determining the concentration of MPRs used in subsequent experiments. After that, the concentration of AAPH-induced oxidative damage in 24 hpf zebrafish embryos was screened.

#### 2.7.1 Determination of zebrafish heart rate

The selected 24 hpf zebrafish embryos were placed in 6-well plates, and the water was replaced once a day. The number of zebrafish heartbeats per minute was counted by microscope after 72 hpf of continuous administration and oxidative induction, and the average value was calculated.

#### 2.7.2 ROS, apoptotic cell staining, and lipid peroxidation staining experiment of zebrafish

Specific fluorescent probes were, respectively, used to stain zebrafish embryos with the purpose of detecting zebrafish cell mortality (acridine orange), intracellular ROS production rate (DCFH-DA), and lipid peroxidation production rate (DPPP). Zebrafish were rinsed with fresh embryonic medium after being incubated for a period of time in a dye-containing medium, then they were anesthetized. After that, we observed with a fluorescence microscope (Leica DMi8, Wetzlar, Germany), and images were taken with a digital camera. Finally, employing Image J software (National Institutes of Health, Bethesda, MD, USA), the fluorescence intensity of individual zebrafish was quantified.

#### 2.7.3 Behavioral analysis

After 96 h of exposure to MRPs in the presence or absence of AAPH, the spontaneous movement of embryos was tracked for 10 min in a 96-well plate at 27.5 ± 1°C between 9 a.m. and 12 a.m. under particularly suitable light conditions. The zebrafish behavior tracking system (Danio Vision, Noldus, Wageningen, Netherlands) was used to record the number of tail coil alternations every 10 min, and the spontaneous movement distance of 7 dpf embryos per minute was calculated.

#### 2.7.4 MDA and GSH assay

The larvae were cleaned three times using pre-cooled PBS, and then 10 larvae were homogenized. After centrifugation at 4°C, based on the manufacturer’s instructions, the levels of malondialdehyde (MDA) and reductive glutathione (GSH) were detected by the assay kit supplied by Nanjing Jiancheng Bioengineering Institute (Nanjing, China).

### 2.8 Preparation and sensory evaluation of condiments

#### 2.8.1 Process optimization of condiments

To obtain high-quality seasonings, the enzymatic hydrolysate was used as raw material to prepare seasoning by adding salt, sodium glutamate, disodium nucleotide, yeast extract, and white granulated sugar. For purpose of exploring the concentration of raw materials in the composite seasoning, we first determined the optimum concentration of each raw material by single factor test. Then, MRPs, salt, white granulated sugar, and sodium glutamate were used as factors to do four-factor and three-level orthogonal experiments to determine the best compounding ratio. The ingredients were mixed in proportion, and the seafood seasoning was obtained after canning and sterilizing.

#### 2.8.2 Sensory evaluation of the condiment

The evaluation of sensory was carried out in the way presented by Fu et al. (31) with minor changes. In the sensory evaluation process, the explanation of the sensory panel should be included: demographic characteristics of the panel, recruitment and training characteristics, and performance analysis results (32).

Select sensory evaluators based on interest, sensitivity, and familiarity with the sample. Sensory evaluators are not allergic to seafood products and pass olfactory and taste sensitivity tests. Sensory training was conducted for sensory evaluators a total of eight times, for about two hours each time, to establish consistent descriptive terms, improve the evaluators’ perception of descriptive words, and standardize the use of sensory scales. In the subsequent simulation test, 10 sensory evaluation team members (5 females and 5 males) were selected based on the difference of no more than 20% between individual team members.

Sensory evaluation indexes included taste, flavor, saltiness, total acceptance and so on. The samples were placed in a 50 mL sanitary cup, and the team members tasted the samples and scored them. Before evaluating each sample, team members were asked to rest for 30 s and were given clean water to rinse their mouths. In each test, 10 team members tested different samples three times.

### 2.9 Statistical analysis

All the data were displayed as means ± SD of three to six separate experiments. GraphPad Prism 8 software (GraphPad Software, San Diego, CA, USA) was applied to conduct statistical analyses using one-way ANOVA. *p* < 0.05 was deemed significant.

## 3 Results

### 3.1 Optimization of enzymatic hydrolysis process of *Urechis unicinctus*

Under the optimum conditions, trypsin, alkaline protease, papain, pepsin, and flavorzyme were used to hydrolyze *Urechis unicinctus*. The three proteases with the highest degree of hydrolysis were trypsin, flavorzyme, and alkaline protease (Supplemenatry Figure S1). Then the simple lattice experimental design was used to obtain the best ratio of complex protease: trypsin: alkaline protease = 21.2: 8.8. Finally, the ultrasonic enzymolysis conditions were determined by the single factor experiment and the response surface experiment. The ultrasonic enzymolysis time was 54.95 min, the ratio of liquid to material was 52: 1, and the amount of enzyme was 3.17%. The optimum enzymatic hydrolysis conditions were that the enzymatic hydrolysis time was 3.16 h, the pH was 8.58, and the enzymatic hydrolysis temperature was 50.48°C. At this time, the degree of hydrolysis (DH) was 34.19%.

### 3.2 Amino acid compositions of *Urechis unicinctus* hydrolysate and *Urechis unicinctus* hydrolysate MRPs

The hydrolysate of *Urechis unicinctus* was modified by the Maillard reaction when mainly amino acids were engaged in the reaction, which would lead to a significant change in amino acid composition. In this experiment, the amino acid composition of *Urechis unicinctus* hydrolysate was established by HPLC before and after the Maillard reaction. The changes in amino acid composition are shown in Table 1, where the total amino acid content of the hydrolysate of *Urechis unicinctus* decreased from 486.65 mg to 310.7 mg after MR, which occurred between reducing sugars and amino acids and gave the food its unique flavor. Studies have shown that bitter amino acids (leucine, lysine, valine, methionine, etc.) were consumed in large quantities during the Maillard reaction (33), so the content of bitter amino acids in the Maillard reaction products was significantly reduced.

**Table 1:** The amino acid composition of *Urechis unicinctus* hydrolysate and *Urechis unicinctus* hydrolysate MRPs.

Amino acid	*Urechis unicinctus* Hydrolysates (mg/g)	Content (%)	*Urechis unicinctus* Hydrolysates MRPs (mg/g)	Content (%)
Asparticacid	56.540 ± 3.852^a^	5.654	37.296 ± 4.176^bc^	3.730
Glutamicacid	72.810 ± 8.481^a^	7.281	47.111 ± 6.178^bc^	4.741
Hydroxy-proline	6.264 ± 0.310^a^	0.626	3.863 ± 0.091^b^	0.386
Serine	25.114 ± 0.223^a^	2.511	15.396 ± 1.020^b^	1.540
Glycine	68.025 ± 1.152^a^	6.803	37.632 ± 2.586^bc^	3.763
Histidine	7.177 ± 0.426^a^	0.718	4.087 ± 0.430^b^	0.409
Arginine	35.365 ± 2.718^a^	3.537	24.702 ± 1.804^b^	2.470
Threonine	15.485 ± 0.562^a^	1.549	10.497 ± 0.365^b^	1.050
Alanine	60.785 ± 5.936^a^	6.079	43.973 ± 3.963^b^	4.397
Proline	15.485 ± 0.497^a^	1.549	10.497 ± 0.238^b^	1.050
Tyrosine	12.425 ± 0.825^a^	1.242	7.710 ± 1.053^b^	0.771
Valine	15.553 ± 0.370^a^	1.555	10.062 ± 0.411^b^	1.006
Methionine	12.012 ± 1.543^a^	1.201	5.346 ± 0.243^b^	0.535
Cystine	0.605 ± 0.062^a^	0.06	0.645 ± 0.087^a^	0.065
Isoleucine	12.546 ± 0.375^a^	1.255	8.106 ± 0.211^b^	0.0811
Leucine	29.416 ± 3.407^a^	2.942	18.724 ± 2.705^b^	1.872
Phenylalanine	13.385 ± 0.753^a^	1.339	8.137 ± 0.621^b^	0.814
Lysine	27.859 ± 0.343^a^	2.786	17.425 ± 4.481^b^	1.742

Different subscript letters in the same line indicate significantly different (*p* < 0.05).

### 3.3 GC–MS analysis of *Urechis unicinctus* hydrolysate and hydrolysate MRPs

GC–MS analysis was employed to study the changes of flavor substances in MRPs of enzymatic hydrolysates and hydrolysates of *Urechis unicinctus*. By comparing with the mass spectrometry database, we speculated on the types of flavor substances contained in the enzymatic hydrolysates and MRPs of enzymatic hydrolysates and analyzed the relative content changes of flavor substances in the enzymatic hydrolysate and the enzymatic hydrolysate MRPs with 2-methyl-3-heptanone as internal standard substance. It can be observed from Table 2 that the content of aldehydes decreased significantly after MR (from 14.21 to 3.33%), among which heptanal, hexanal, octanal, and 2-methyl-2-pentenal all decreased to 0%, nonanal decreased from 3.42 to 0.89%, and acetaldehyde decreased from 2.84 to 0.32%. The content of ketones also decreased (from 6.2 to 1.27%), especially acetone, nonanone, and undecane, which decreased to 0%.

**Table 2:** Comparison of flavor substances between *Urechis unicinctus* hydrolysate and hydrolysate MRPs.

Category	Compound	*Urechis unicinctus* hydrolysates			*Urechis unicinctus* Hydrolysates MRPs		
		Content (μg/g)	Percentage (%)	SI	Content (μg/g)	Percentage (%)	SI
Alcohol	1-Octen-3-ol	63.11 ± 1.17^a^	5.88	88	66.66 ± 2.26^a^	6.21	91
	Ethyl alcohol	5.15 ± 0.12^c^	0.48	92	56.03 ± 0.19^d^	5.22	87
	Cyclopentyl alcohol	9.55 ± 0.69^a^	0.89	88	28.02 ± 0.60^c^	2.61	90
	tert-Butyl alcohol	16.10 ± 0.65^c^	1.50	96	22.86 ± 0.67^b^	2.13	97
	1-Pentene-3-ol	3.76 ± 0.26^c^	0.35	91	23.08 ± 0.35^a^	2.15	93
	2-Pentene-1-ol	2.47 ± 0.24^c^	0.23	87	18.14 ± 0.26^a^	1.69	90
	Isopropanol	3.11 ± 0.12^a^	0.29	97	3.76 ± 0.21^a^	0.35	95
	Diphenylsilanediol	11.16 ± 0.65^c^	1.04	89	4.72 ± 0.31^b^	0.44	90
	N-amyl alcohol	3.11 ± 0.38^a^	0.29	96	3.33 ± 0.12^a^	0.31	87
	Methanol	14.28 ± 0.71^b^	1.33	99	15.24 ± 0.57^b^	1.42	95
	Phenylethyl alcohol	23.08 ± 1.82^c^	2.15	93	31.99 ± 2.17^b^	2.98	88
	Furfuralcohol	22.65 ± 1.69^a^	2.11	96	25.44 ± 1.98^a^	2.37	94
	Sum	177.54 ± 8.50	16.54		299.26 ± 9.69	27.88	
Acid	Adipic acid	25.55 ± 1.13^c^	2.38	89	33.38 ± 1.36^b^	3.11	95
	Caproic acid	8.37 ± 0.27^a^	0.78	87	14.81 ± 0.40^b^	1.38	93
	Pelargonic acid	37.89 ± 1.86^c^	3.53	95	45.30 ± 2.09^c^	4.22	92
	Alanine	9.66 ± 0.11^b^	0.90	94	6.23 ± 0.34^b^	0.58	87
	Fumaric acid	1.29 ± 0.05^a^	0.12	91	9.45 ± 0.28^b^	0.88	96
	Acetic acid	102.29 ± 3.57^a^	9.53	97	130.52 ± 3.82^b^	12.16	96
	Propionic acid	41.54 ± 2.24^c^	3.87	92	53.36 ± 2.47^b^	4.99	97
	Oleic acid	14.60 ± 0.45^a^	1.36	97	32.42 ± 0.68^b^	3.02	94
	Myristic acid	7.30 ± 0.17^b^	0.68	91	14.60 ± 0.20^a^	1.36	88
	Palmitic acid	10.30 ± 0.31^a^	0.96	86	18.78 ± 0.45^b^	1.75	91
	Benzoic acid	2.58 ± 0.06^a^	0.24	90	8.16 ± 0.29^b^	0.76	86
	Phenylacetic acid	6.01 ± 0.12^c^	0.56	97	28.87 ± 0.34^a^	2.69	99
	Sum	267.38	24.91		396.08	36.90	
Aldehyde	Heptanal	14.17 ± 0.38^a^	1.32	96	-	-	
	Hexanal	10.20 ± 0.54^a^	0.95	95	-	-	
	Acetaldehyde	30.48 ± 1.02^c^	2.84	93	3.43 ± 0.07^a^	0.32	96
	Benzaldehyde	20.72 ± 0.72^a^	1.93	97	5.96 ± 0.10^b^	0.53	96
	Heptanal	9.23 ± 0.30^a^	0.86	96	-	-	
	Octanal	11.81 ± 0.41^c^	1.10	97	-	-	
	Nonanal	36.71 ± 1.33^c^	3.42	96	9.55 ± 0.21^b^	0.89	96
	2-Methyl-2-pentenal	13.42 ± 0.27^a^	1.25	90	-	-	
	Butylaldehyde	5.80 ± 0.09^c^	0.54	91	17.07 ± 0.19^b^	1.59	93
	Sum	152.53	14.21		35.74	3.33	
Ketone	2,6-dimethyl-4-pyranone	1.40 ± 0.08^a^	0.13	87	6.23 ± 0.09^b^	0.58	90
	2-Heptanone	3.11 ± 0.11^a^	0.29	91	7.94 ± 0.13^b^	0.74	86
	1-Pentene-3-one	13.52 ± 0.19^c^	1.26	96	3.86 ± 0.07^b^	0.36	92
	Butanone	9.88 ± 0.15^c^	0.92	98	1.82 ± 0.03 ^b^	0.17	99
	Acetone	9.23 ± 0.43^a^	0.86	87	-	-	
	Nonanone	10.41 ± 0.28^a^	0.97	82	-	-	
	Undecanone	20.39 ± 0.79^c^	1.90	95	-	-	
	Sum	66.55	6.20		13.63	1.27	
Hydrocarbon	Dodecane	21.25 ± 0.73a	1.98	95	3.22 ± 0.17^c^	0.30	83
	Tetradecane	28.77 ± 1.01^c^	2.68	96	3.65 ± 0.11^a^	0.34	95
	Hexadecane	27.48 ± 0.36^a^	2.56	94	2.79 ± 0.02^c^	0.26	91
	Heptadecane	1.50 ± 0.03^c^	0.14	92	4.94 ± 0.10^c^	0.46	95
	Eicosane	12.67 ± 0.12^c^	1.18	96	5.37 ± 0.06^c^	0.50	93
	Styrene	48.73 ± 3.43^a^	4.54	99	-	-	
	Tridecane	21.47 ± 0.81^c^	2.00	84	-	-	
	Pentadecane	19.54 ± 0.63^a^	1.82	89	-	-	
	Tetracosane	6.76 ± 0.21^c^	0.63	91	4.83 ± 0.08^c^	0.45	89
	Sum	188.16	17.53		24.79	2.31	
Phenol	Maltol	-	-		57.10 ± 1.98^c^	5.32	99
	5-Methyl-2-isopropylphenol	-	-		23.94 ± 0.76^c^	2.23	97
	2,4-Di-tert-butylphenol	-	-		1.61 ± 0.03^a^	0.15	90
	Sum		0.00		82.65	7.70	

Different subscript letters in the same line indicate significantly different (*p* < 0.05). “-” means not detected.

The content of acid compounds raised after the Maillard reaction (from 24.91 to 34.9%), and the proportion of acid compounds contained in the enzymatic hydrolysate increased to a certain extent. Alcohols also increased from 16.54 to 27.88%, and the most significant change was ethanol (from 0.48 to 5.22%). In addition, phenolic compounds were achieved from nothingness to being, maltol (from 0 to 5.32%) and 5-methyl-2-isopropylphenol (from 0 to 2.23%) increased significantly.

### 3.4 Determination of antioxidant activity

**Figure 1:**
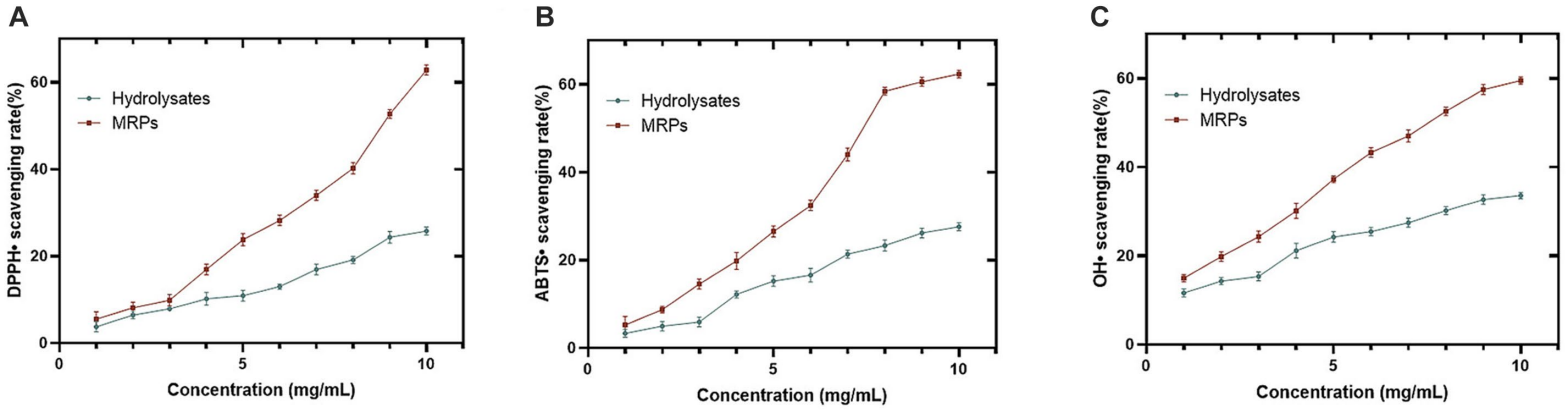
Comparison of antioxidant ability between *Urechis unicinctus* hydrolysate and hydrolysate MRPs. DPPH• **(A)**, ABTS• **(B)**, and OH• **(C)** scavenging activity of hydrolysate and hydrolysate MRPs. n = 3, Data are expressed as mean ± SD.

The antioxidant properties of MRPs and *Urechis unicinctus* hydrolysate were determined by three methods, and it was observed that the scavenging ability of hydrolysates enhanced after MR and showed a concentration dependence (Figures 1A–C). In addition, it was shown that the difference between the antioxidant property of MRPs and the hydrolysate increased with growing concentration. For example, the enzymatic digest of 4 mg/mL showed no apparent difference after MR, and then it became larger. The results obtained by the three methods are consistent with each other, so they can be good proof of the enhanced antioxidant activity of the hydrolysate after the Maillard reaction.

### 3.5 MRPs attenuate of AAPH-induced oxidative damage in L_02_ cells

**Figure 2:**
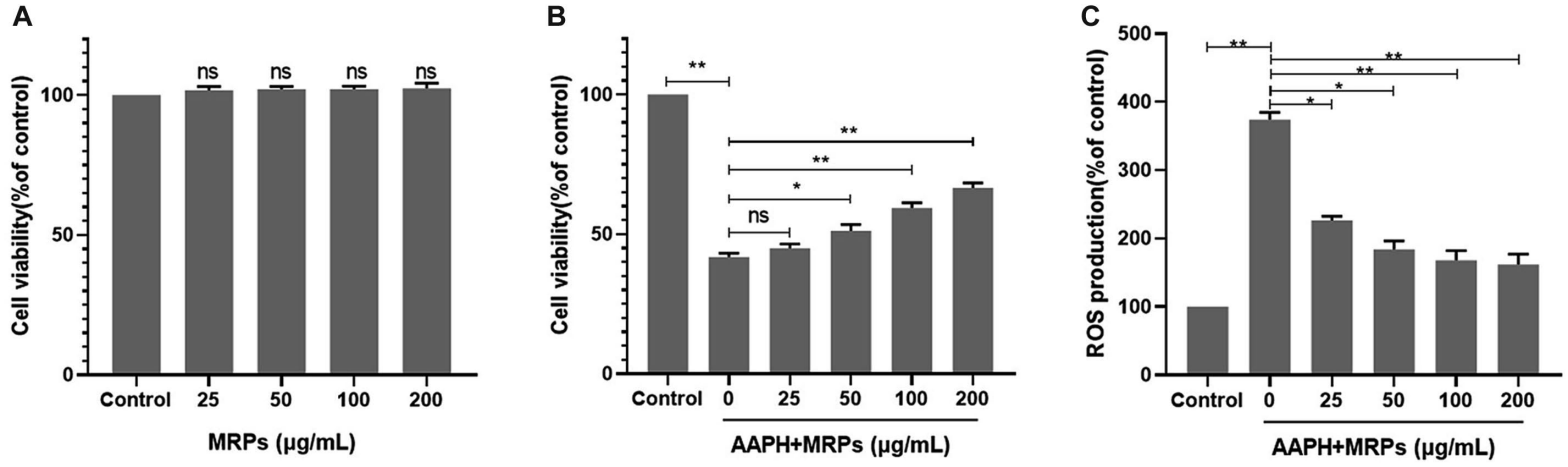
MRPs attenuated AAPH-induced cell injury and ROS generation in L_02_ cells. **(A)** MTT assay to verify whether different concentrations of MRPs (25, 50, 100, 200 μg/mL) had cytotoxicity. **(B)** MRPs were added 1 h before AAPH. Effect of MRPs on AAPH-induced cell viability. **(C)** Effect of MRPs on the ROS levels of AAPH-induced L_02_ cells. n = 4, Data are expressed as mean ± SD. * *p* < 0.05, ** *p* < 0.01 vs. AAPH group, ns, not significant.

To verify the antioxidant activity of the *Urechis unicinctus* hydrolysate MRPs, we established an AAPH-induced damage model based on previous studies (34, 35). AAPH is a free radical promoter that disintegrates at 37°C to form two free radicals with carbon atoms as the core. In the presence of oxygen, this triggers a free radical chain reaction to generate ROS, leading to cell damage. Cell viability after treatment with varying concentrations of MRPs was first measured using the MTT assay. MRPs did not show cytotoxicity at concentrations below 200 μg/mL compared to the control group (Figure 2A). MRPs at non-toxic concentrations were utilized in subsequent experiments to detect their protective effects against oxidative damage. Cells were incubated with varying doses of MRPs samples (0, 25, 50, 100, and 200 μg/mL) for 1 h, preceded by the addition of 15 μM AAPH for 16 h. As shown in Figure 2B, cell viability was drastically decreased in the AAPH group, and pretreatment of MRPs led to a positive response to the cell viability of oxidative stress injury in a concentration-dependent manner. Particularly, the cell viability treated with 200 μg/mL MRPs was markedly raised compared to the AAPH group.

**Figure 3:**
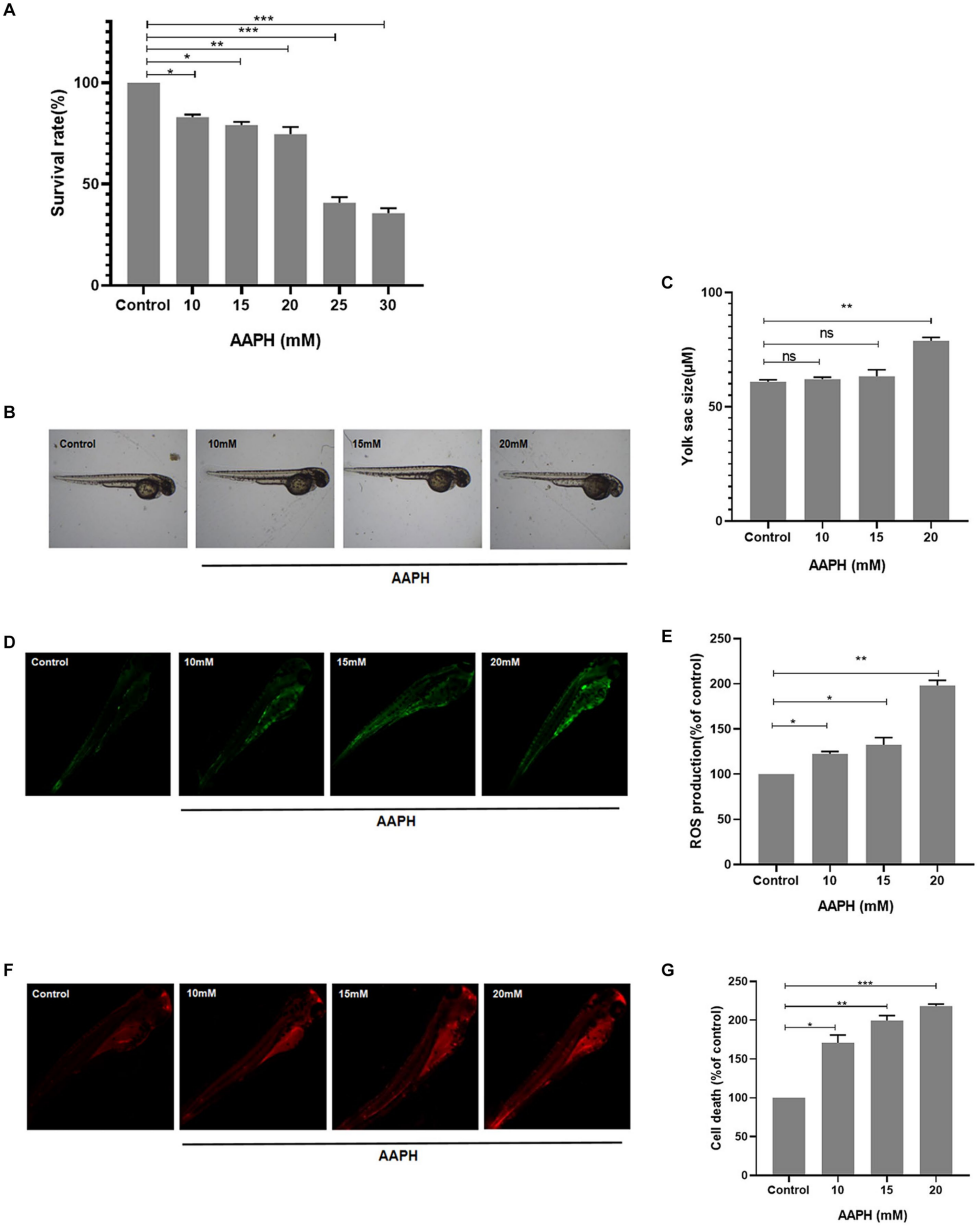
Construction of AAPH-induced oxidative stress model *in vivo*. The effects of different concentrations of AAPH on the survival rate **(A)**, yolk sac size **(B,C)**, ROS production rate **(D,E)** and cell death rate **(F,G)** of zebrafish. *n* = 10, Data are presented as means ± SD and analyzed by one-way ANOV A followed. * *p* < 0.05, ** *p* < 0.01 vs. control, ns, not significant.

In addition, endo cellular ROS generation was assessed employing DCFH-DA fluorescence staining. In Figures 3B,C, the fluorescence intensity of the AAPH-exposed group was 98,963, which was dramatically higher than that in the control group. As expected, pretreatment with different dosages of MRPs remarkably reduced ROS overaccumulation in a dose-dependent manner, with fluorescence intensities reduced to 311,060, 253,936, 232,131, and 223,833, respectively. The results suggest that *Urechis unicinctus* hydrolysate MRPs can protect L_02_ cells by attenuating oxidative damage. The *Urechis unicinctus* hydrolysate MRPs could be a potential biomolecular candidate to inhibit cell oxidative stress and ROS generation.

### 3.6 Protective effect of MRPs On oxidative damage *in vivo*

#### 3.6.1 Construction of AAPH oxidative damage model *in vivo*

The antioxidant properties of an active substance can be verified by *in vitro* chemical methods, but these methods are not sufficient to support whether the substance has antioxidant properties *in vivo*. Therefore, an *in vivo* model of zebrafish was further used to investigate the protective impact of MRPs on AAPH-induced oxidative strain. The mortality rate of zebrafish embryos increased with increasing AAPH concentration in a dose-dependent manner. The survival rate of zebrafish embryos decreased to less than 50% when induced with 25 mM and 30 mM doses and was unsuitable for further index testing (Figure 3A). Zebrafish yolk sac size also increased with increasing AAPH concentration, showing distinct enlargement at 20 mM (Figure 3B). The fluorescence detection results of cellular ROS formation and cell death in zebrafish showed that AAPH induction could increase the ROS level in zebrafish, and the ROS generation rate was higher with increasing induction concentration (Figures 3B,C). The cell death rate of zebrafish also increased sharply with the increase of concentration (Figure 3D). In summary, we selected 20 mM AAPH to establish a zebrafish oxidative stress induction model for subsequent evaluation of the antioxidant property of MRPs.

#### 3.6.2 Screening of MRPs concentration In zebrafish

**Figure 4:**
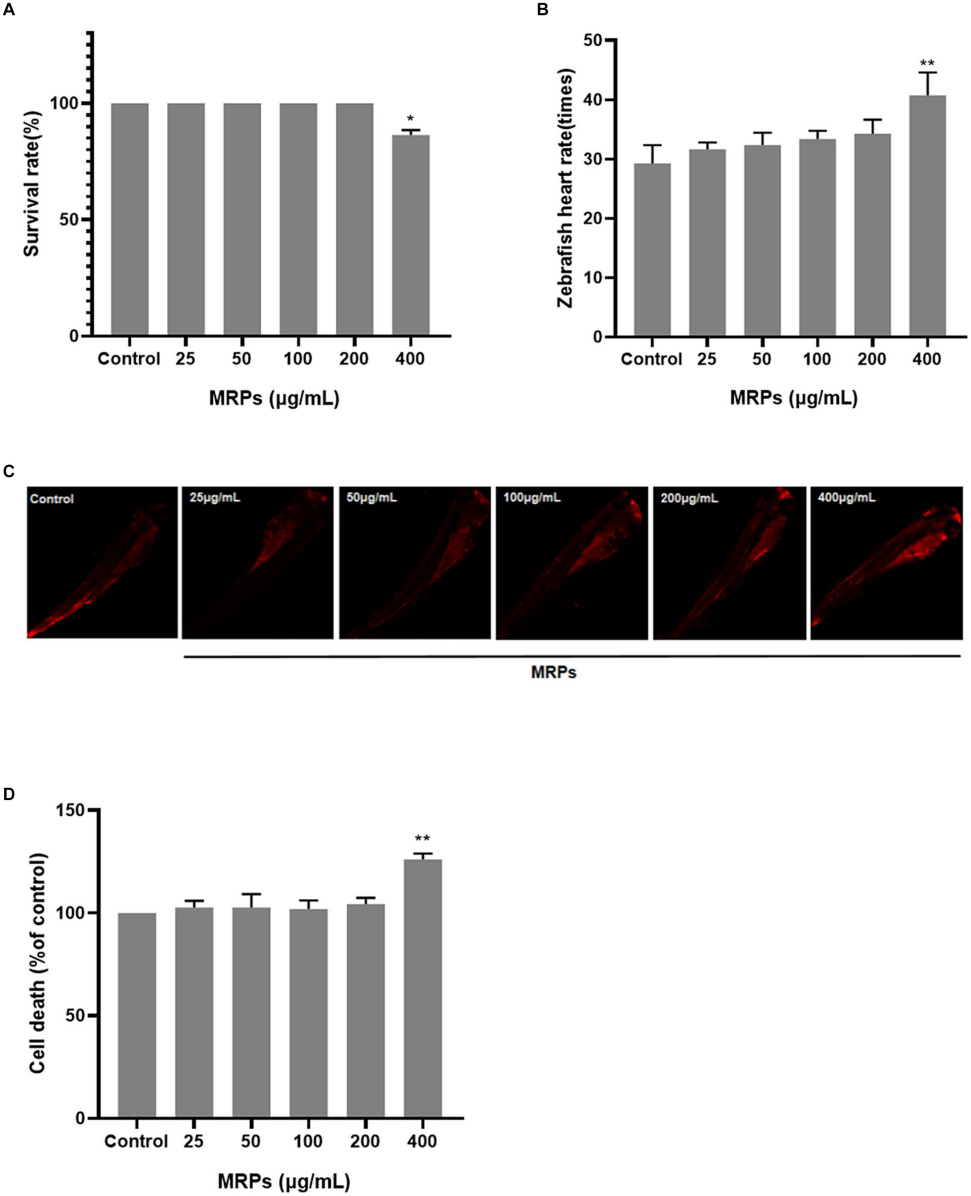
Screening of MRPs concentration. The effects of different concentrations of MRPs on the survival rate **(A)**, heart rate **(B)** and cell death rate of zebrafish **(C,D)**. *n* = 10, Data are presented as means ± SD and analyzed by one-way ANOV A followed. * *p* < 0.05, ** *p* < 0.01 vs. control, ns, not significant.

The following methods were utilized to observe the impact of MRPs at various doses (25, 50, 100, 200 and 400 μg/mL) on the viability of zebrafish. MRPs in the dose range of 0–200 μg/mL had little effect on the zebrafish embryo survival rate, and the survival rate decreased when the concentration attained 400 μg/mL (Figure 4A). The heartbeat rate of zebrafish was significantly increased at a dose of 400 μg/mL compared to the control group (Figure 4B). As demonstrated in Figures 4B,C, in comparison with the control group, there was no obvious change in zebrafish cell mortality after treatment with MRPs below 200 μg/mL, while MRPs at 400 μg/mL caused a significant increase. Therefore, 25, 50, 100, and 200 μg/mL of MRPs were chosen for the subsequent experiments.

#### 3.6.3 Protective effects of MRPs against AAPH-induced oxidative stress in zebrafish

**Figure 5:**
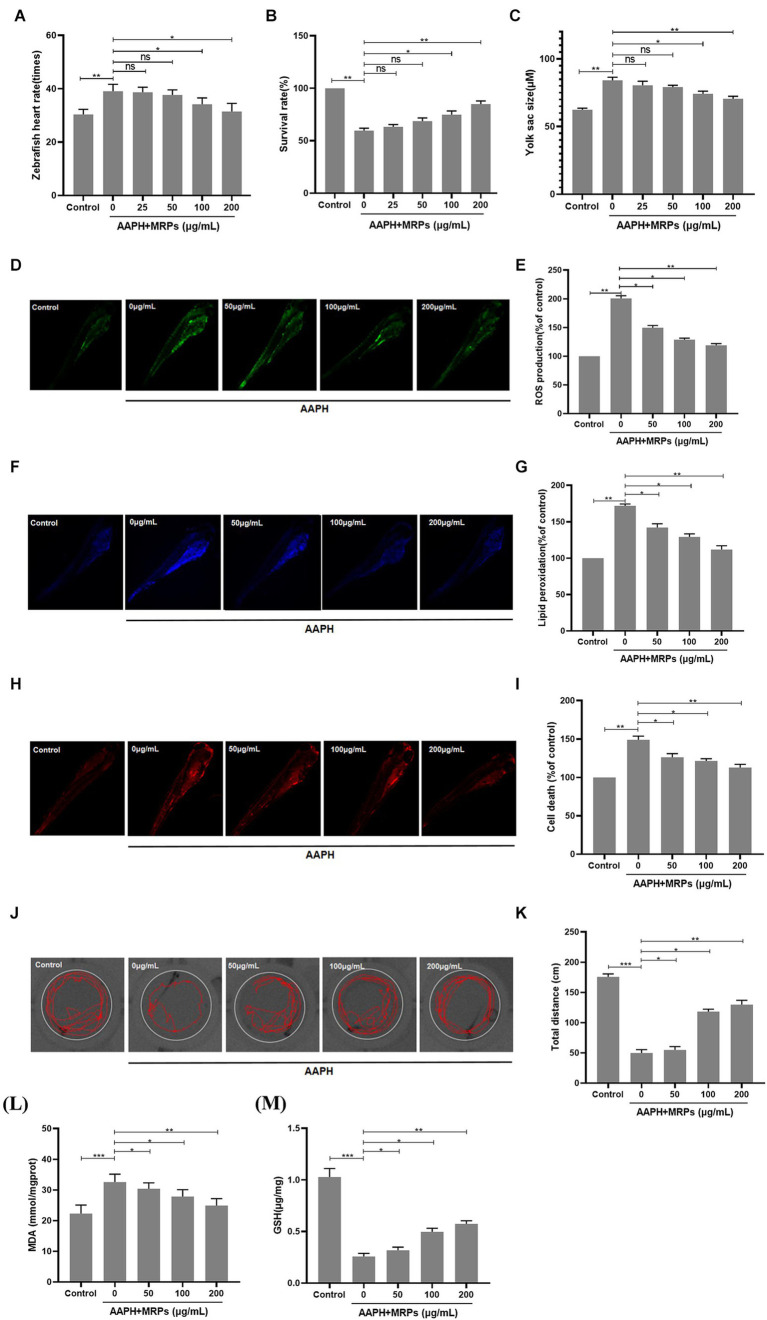
Protective effects of MRPs against oxidative stress in zebrafish model. Effect of MRPs on the heart rate **(A)**, survival rate **(B)**, and yolk sac size **(C)** in AAPH-induced zebrafish embryos. Inhibitory effect of MRPs on AAPH-induced ROS production **(D,E)**, lipid peroxidation **(F,G)**, and cell death rate **(H,I)** in zebrafish embryos. They were measured after staining with DCFH-DA, DPPP, and acridine orange followed by image analysis and fluorescence microscopy. **(J,K)** Distance traveled of zebrafish larvae at 96 hpf after exposure to MRPs absence or presence of AAPH. Effect of MRPs on MDA **(L)** and GSH **(M)** in zebrafish. *n* = 10, Data are presented as means ± SD and analyzed by one-way ANOV A followed. * *p* < 0.05, ** *p* < 0.01 and *** *p* < 0.001 vs. AAPH group, ns, not significant.

Oxidative injury may eventually result in cell death, overproduction of ROS and lipid peroxidation. In this study, the improved impacts of MRPs against AAPH-induced cell death, reduced locomotor activity, ROS production, and lipid peroxidation in zebrafish were examined. As indicated in Figures 5A,B), in comparison with the model group, zebrafish embryo survival and cell mortality were markedly reduced when MRPs were administered in a dose-dependent manner. The zebrafish heart rate and zebrafish yolk sac size could reflect the survival status of zebrafish. Treatment with MRPs reversed the increased heart rate and enlarged yolk sac size of zebrafish caused by AAPH. After treatment with only AAPH, the total distance of zebrafish movement was obviously decreased. However, MRPs showed a good improvement in locomotor activity in zebrafish (Figures 5B,C). The effects of MRPs on AAPH-induced ROS production and lipid peroxidation levels are indicated in Figures 5D,E), respectively. When zebrafish embryos were treated with MRPs in advance of AAPH treatment, a dose-dependent reduction in ROS and lipid peroxidation production was observed. Moreover, MRPs pretreatment ameliorated the increased MDA content and reduced GSH content in zebrafish caused by AAPH treatment (Figures 5F,G). These data indicated that MRPs pretreatment inhibited ROS generation and lipid peroxidation to protect zebrafish from oxidative injury.

### 3.7 Preparation of seasonings

**Figure 6:**
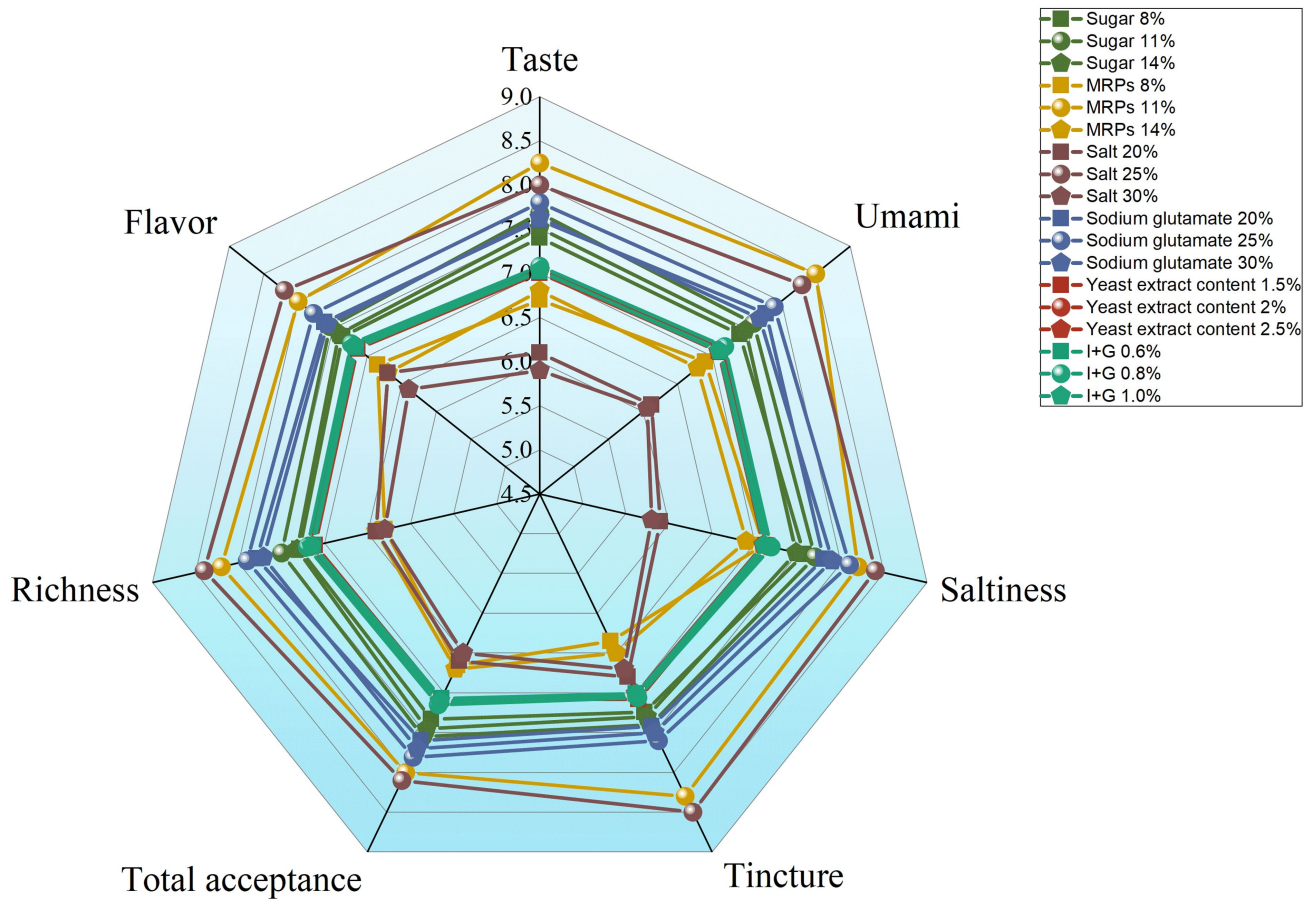
Effects of different raw materials on the flavor of *Urechis unicinctus* MRPs seasonings.

There is no doubt that the most critical factor in seasoning is flavor. The single-factor experiment showed that the change of I + G and yeast extract content had little effect on the sensory scores. The main and secondary factors affecting the sensory score were salt, MRPs, sodium glutamate, and white sugar (Figure 6). The content of I + G was 0.8%, and yeast extract was 1.5% for subsequent experiments. With MRPs, salt, white sugar, and sodium glutamate as factors, the orthogonal experiment of four factors and three levels (Table 3) was conducted to determine the best formula process. The data showed that the optimal combination was MRPs (11%), sugar (11%), salt (25%), sodium glutamate (25%), I + G (0.8%), and yeast extract (1.5%) (Table 4). Finally, a high-end seasoning with hygienically qualified, white powder, unique seafood flavor, and pleasant taste was obtained.

**Table 3:** The orthogonal test factors and levels.

Levels	Factors			
	A MRPs (%)	B white sugar (%)	C salt (%)	D sodium glutamate (%)
1	8	8	20	20
2	11	11	25	25
3	14	14	30	30

**Table 4:** The result of Orthogonal experimental.

Group	Factors				Sensory scores
	A	B	C	D	
1	1	1	1	1	6.15
2	1	2	2	2	8.54
3	1	3	3	3	6.2
4	2	1	2	3	8.02
6	2	3	1	2	7.12
7	3	1	3	2	6.86
8	3	2	1	3	6.34
9	3	3	2	1	7.78
K1	6.963	7.010	6.537	6.943	
K2	7.347	7.260	8.113	7.507	
K3	6.993	7.033	6.653	6.853	
R	0.384	0.250	1.576	0.654	

## 4 Discussion

With the improvement of people’s living standards, aquatic condiments with health care functions are becoming more popular (2). *Urechis unicinctus*, also known as “naked sea cucumber,” has the potential to prepare seafood condiments because of its low fat and high protein content. However, the hydrolysate of *Urechis unicinctus* has a bitter taste that is unique to seafood. MR can improve the flavor and color of food and in some cases, can enhance protein functionality (36, 37). Therefore, we attempted to use the enzymatic hydrolysate of *Urechis unicinctus* for MR with glucose and found that the bad flavor of the enzymatic hydrolysate was improved after the reaction. MRPs showed prosperous antioxidant capacity and the ability to alleviate oxidative damage *in vitro* and *in vivo*. Then, an umami-rich, nutritious, and healthy seasoning was prepared from the Maillard reaction product of *Urechis unicinctus* hydrolysate.

*Urechis unicinctus* has the potential to develop functional seafood seasonings due to its rich protein content, reasonable amino acid composition, and biological activities such as antioxidant and hypoglycemic. However, the enzymatic hydrolysate of *Urechis unicinctus* has the characteristic fishy flavor of aquatic products. Therefore, improving this unsatisfactory flavor has become the key to the preparation of *Urechis unicinctus* seasoning. The Maillard reaction treatment of aquatic product protein hydrolysates can achieve the purpose of deodorization and aroma enhancement, which is favored by the food industry. Therefore, firstly, in this study, the hydrolysate was reacted with glucose at 120°C for 2 h to obtain the MRPs of *Urechis unicinctus* hydrolysate. The total amino acid content decreased after the reaction, which was to be attributed to the fact that MR takes place mainly between reducing sugars and amino acids, giving the food a unique flavor (7, 37). Among them, leucine, lysine, methionine, valine, and other amino acids that produce a bitter taste (38, 39) were apparently reduced, which was similar to the findings obtained by Lan and Liu et al. in the study of the xylose-soybean peptide maillard reaction system (40). Hexanal, heptanal, octanal, nonanal, etc., which are responsible for the fishy smell of seafood (41, 42), were markedly reduced after MR, which was consistent with the study of Zhao et al. (43). After MR, the content of short-chain fatty acids such as acetic acid and propionic acid increased, and their thresholds were lower, which had a cheese flavor and could play a certain modification role. Additionally, the content of acetic acid and propionic acid increased after MR. Alcohols are mainly derived from amino acid reduction and fat oxidation, which usually have plant fragrances (44). Phenolic compounds are beneficial in encouraging the release of fishy odor components, reducing the fishy odor of aquatic products significantly (45), and harmonizing the overall flavor of the food. In summary, the findings indicated that MR enhanced the flavor of the hydrolysates of *Urechis unicinctus*.

Simultaneously, the MRPs showed more competitive free radical scavenging capacity in contrast to that of the hydrolysates. We speculated that the increase in free radical scavenging capacity might be related to changes in the composition of the hydrolysates, especially the increase in acids and phenolics in the MRPs. The antioxidant activity of phenolics and acids has been widely reported. Dini et al. (46) added 5-methyl-2-isopropyl phenol to polylactic acid at a certain concentration, and found that its antioxidant capacity in water was enhanced. Wang et al. (47) found that the antioxidant property of chitosan modified by fumaric acid increased from 63 to 85% in ionic liquid solution by the DPPH free radical scavenging method. Furthermore, oyster protein hydrolysate showed superior free radical scavenging ability due to the increased phenolic and acid content after MR (48). Therefore, we concluded that MR improved the antioxidant properties of the hydrolysates, and the change in composition was essential for the increase in antioxidant capacity.

L_02_ cells have been validated as an ideal cellular model for evaluating antioxidant activity (49, 50). For the sake of further verifying the antioxidant capacity of MRPs, we utilized L*_02_* cells to evaluate their intracellular antioxidant efficacy. AAPH is a free radical initiator applied extensively in mimicking oxidative stress states (51). Therefore, AAPH was employed in this study to induce oxidative strain L_02_ cells. The viability of L_02_ cells exposed to AAPH was markedly reduced. This might be due to the fact that cellular damage attributed to ROS-induced oxidative stress usually impairs biomolecular functions and results in cell death (52, 53). However, cell death was inhibited with MRPs, indicating that MRPs can protect cells from AAPH-induced cytotoxicity. We utilized an oxidant-sensitive DCFH-DA fluorescence sensor to quantify the level of ROS generation in cells and determined that MRPs block AAPH-induced ROS generation and subsequent oxidative stress. These findings demonstrated that MRPs played a protective role in ROS-induced oxidative stress, thereby reducing cellular damage.

In accordance with recent reports, zebrafish can be utilized as a quick and straightforward model to evaluate anti-oxidative stress activity *in vivo* (34, 35). As a consequence, in this research, we made use of the zebrafish oxidative damage model to explore the antioxidant effect of MRPs *in vivo*. The outcomes indicated that AAPH dramatically raised cell death and ROS levels in zebrafish embryos. In contrast, MRPs suppressed this phenomenon. In the research, AAPH treatment significantly increased lipid peroxidation in zebrafish embryos, which was due to the fact that lipid peroxidation might be a type of cellular harm brought on by free radicals (54). However, MRPs effectively inhibited the formation of this lipid peroxidation. Accordingly, MRPs also exhibited satisfactory effects in inhibiting ROS overproduction and attenuating oxidative damage in a zebrafish model *in vivo*, further validating the antioxidant capacity and nutritional value of MRPs.

As stated previously, MRPs of the *Urechis unicinctus* hydrolysates showed strong aroma and favorable antioxidant effect, which were high-quality raw materials for preparing functional seafood seasonings. Consequently, we utilized MRPs as the raw material to determine the best formula by process optimization and sensory evaluation, and a high-end functional seasoning with a strong aroma and the antioxidant effect was prepared.

## 5 Conclusion

In summary, the Maillard reaction can improve the flavor of *Urechis unicinctus* hydrolysate and enhance its antioxidant property. Next, the MPRs of *Urechis unicinctus* hydrolysates can be utilized for industrial production of functional seafood seasonings and also provide an effective way to realize the high-value utilization of marine resources.

## Data availability statement

The original contributions presented in the study are included in the article/Supplementary material, further inquiries can be directed to the corresponding author.

## Ethics statement

The animal study was approved by Experimental Animal Ethics Committee of Yantai University. The study was conducted in accordance with the local legislation and institutional requirements.

## Author contributions

MD: Conceptualization, Writing – original draft, Formal analysis, Methodology, Software, Validation. WY: Data curation, Formal analysis, Methodology, Resources, Validation, Writing – original draft. ND: Formal analysis, Software, Writing – original draft. MJ: Investigation, Visualization, Writing – original draft. YC: Funding acquisition, Project administration, Resources, Supervision, Writing – review & editing. JG: Funding acquisition, Project administration, Resources, Supervision, Writing – review & editing.
