# Supplementary figures and images for: Antioxidant, aroma, and sensory characteristics of Maillard reaction products from Urechis unicinctus hydrolysates: development of food flavorings
Source: Front Nutr. 2024 Feb 6;11:1325886. doi: 10.3389/fnut.2024.1325886 (PMC10876865; doi:10.3389/fnut.2024.1325886)

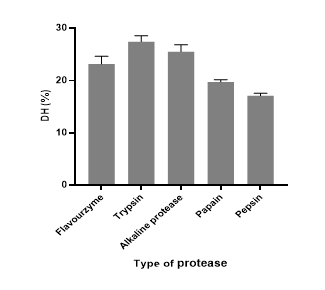

Supplement: Supplementary file 1 [file Image_1.JPEG]
